# On measuring selection in experimental evolution

**DOI:** 10.1098/rsbl.2010.0580

**Published:** 2010-09-01

**Authors:** Luis-Miguel Chevin

**Affiliations:** Division of Biology, Imperial College London, Silwood Park, Buckhurst Road, Ascot SL5 7PY, UK

**Keywords:** mutation fitness effects, experimental evolution, population growth, density dependence, frequency dependence

## Abstract

Distributions of mutation fitness effects from evolution experiments are available in an increasing number of species, opening the way for a vast array of applications in evolutionary biology. However, comparison of estimated distributions among studies is hampered by inconsistencies in the definitions of fitness effects and selection coefficients. In particular, the use of ratios of Malthusian growth rates as ‘relative fitnesses’ leads to wrong inference of the strength of selection. Scaling Malthusian fitness by the generation time may help overcome this shortcoming, and allow accurate comparison of selection coefficients across species. For species reproducing by binary fission (neglecting cellular death), ln2 can be used as a correction factor, but in general, the growth rate and generation time of the wild-type should be provided in studies reporting distribution of mutation fitness effects. I also discuss how density and frequency dependence of population growth affect selection and its measurement in evolution experiments.

## 1. Introduction

The aim of this opinion piece is to clarify the definition and measurement of selection coefficients in evolution experiments investigating fitness effects of mutations. Such studies, from the accumulation of deleterious mutations under relaxed selection over many generations [1–3] to the identification of spontaneous or induced mutations as they arise [4–7], have generated considerable interest recently (reviewed in [8,9]), notably because of their important bearings on many questions across evolutionary genetics. First, they allow testing theoretical predictions about the genetics of adaptation, such as the size of beneficial mutations that get fixed in a population, or the cost of complexity for adaptation (reviewed in [10]). Second, they uncover the intensity of deleterious mutation effects, enabling quantitative predictions to be made about, e.g. the evolution of recombination [11], of selfing [12], or the extinction of small populations [13]. Third, they allow us to address fitness interactions between genes (epistasis) and the ruggedness of fitness landscapes [14–16], which determines the very nature of the evolutionary process from a genetic standpoint. Here I argue that one of the most popular measurements of fitness in evolution experiments (the ratio of Malthusian parameters) does not have a clear evolutionary meaning, and should be replaced by a measurement relating more directly to the dynamics of frequency change of mutations.

## 2. Fitness and selection

The primary goal of measuring fitness effects of mutations is to relate them to the evolutionary dynamics of alleles under selection. This is quantified by the selection coefficient *s*. Consider an asexual (haploid) population consisting of two genotypes, a mutant and a wild-type, with population sizes (or density) *M* and *N*, and frequencies *p* and (1 − *p*). With continuous growth and no age structure, the selection coefficient can be defined as2.1
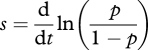
[17], which has units of time^−1^. The frequency of the mutant increases if *s* > 0 and decreases if *s* < 0, at a speed determined by *s*. Since the ratio of allelic frequencies is also the ratio of numbers (or densities) of each genotype, we may also write2.2
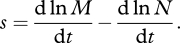


In particular, if selection is density independent and there are no interactions between genotypes, then2.3
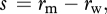
where *r* is the Malthusian parameter [17] or intrinsic rate of increase of each genotype (m, mutant; w, wild-type).

**Figure 1.**
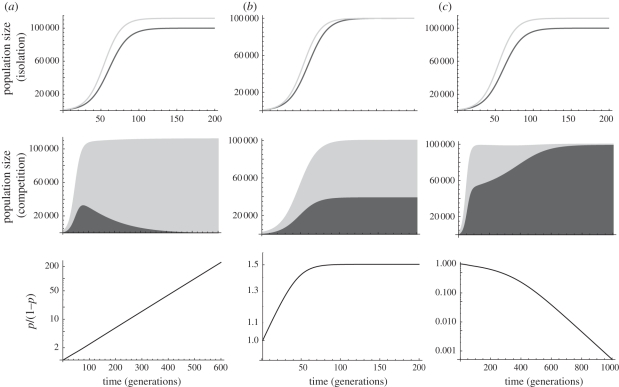
Selection and demography in isolation versus competition. The population sizes of two genotypes grown in isolation (first row: dark grey, wild-type; light grey, mutant) or in competition (second row: dark grey area, wild-type; light grey area, mutant) are shown together with the ratio of genotypic frequencies in competition (third row: note the logarithmic scale on the *y*-axis) for three demographic scenarios. Scenario (*a*) leads to frequency- and density-independent selection. Scenario (*b*) illustrates frequency-independent but density-dependent selection, in the particular case where both genotypes have the same carrying capacity *K*. In this case, *s* tends to 0 as the population approaches the carrying capacity. In scenario (*c*), selection is density-independent but frequency-dependent. Recursions were made from the discrete-generation model in equation (A2) from the electronic supplementary material, appendix, with logistic population growth, using *λ*_w_ = 1.08, *λ*_m_ = 1.09, *K*_w_ = 100 000 in all scenarios, and *K*_m_ = *K*_w_ ln(*λ*_m_)/ln(*λ*_w_) and *ι*_w_ = *ι*_m_ = 1 in (*a*); *K*_m_ = *K*_w_ and *ι*_w_ = *ι*_m_ = 1 in (*b*); *K*_m_ = *K*_w_ ln(*λ*_m_/*λ*_w_), *ι*_w_ = 1 and *ι*_m_ = 0.98 in (*c*).

In practice, *r* is estimated as the regression slope of log-population size against time in the exponential phase, that is, at low population density (assuming no Allee effects). Note that *selection* can be density independent even if *population growth* is density dependent, but this implies that the genotype which initially grows faster also has a higher carrying capacity (figure 1*a*, first row; electronic supplementary material, appendix; see also [18,19]). When this is valid, *s* is constant and from equation (2.1), the ratio of genotypic frequencies increases exponentially in time (figure 1*a*, second row), resulting in a logistic trajectory of the mutant frequency in time. Moreover, in this case, equation (2.3) holds whether each *r* is measured from cultures of each genotype in isolation, or in a competition experiment mixing both genotypes. However, when in competition, more statistical significance can be obtained by estimating *s* directly from equation (2.1) [20].

If selection is density dependent, the selection coefficient changes with population size (figure 1*b*), so that measuring the growth rate in the exponential phase is not sufficient to estimate *s*. Similarly, with genotype-by-genotype interactions in fitness, *s* changes with the relative frequencies of each genotype (figure 1*c*). Disentangling these two possible causes of changes in *s* (density dependence and frequency dependence) requires combining a competition experiment with cultures of each genotype in isolation (electronic supplementary material, appendix).

Most population genetic models are formulated in discrete non-overlapping generations rather than continuous ones as above, and in this case, an equivalent to equation (2.1) is2.4
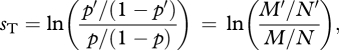
with primes denoting values in the next generation, and the subscript T indicating that the evolutionary change is taken over a generation. In this case, if selection is density independent and there are no genotype-by-genotype interactions, then2.5
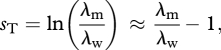
with *λ*_m_ and *λ*_w_ the density-independent components of the per-generation multiplicative growth rate of each genotype (electronic supplementary material, appendix). With overlapping (but discrete) generations, such that the population is structured by ages, or if life stages can be identified (such as larva, juvenile, adult), equation (2.5) is also valid for asexuals (and can be used as a weak-selection approximation for sexuals) if **λ** is the leading eigenvalue of the projection matrix of transition rates (juvenile/adult survival and fecundity) [21,22, p. 61].

Hence, with discrete generations, the ratio *λ*_m_/ *λ*_w_ of the fitness of the mutant to that of the wild-type (or ‘relative fitness’, unitless) determines the evolutionary dynamics. Note that **λ** is necessarily positive (and the population size decreases if **λ** < 1), while *r* can be negative for a decreasing population. Because a negative *r* cannot be measured starting from a small population, some authors have pooled all mutants with (unmeasured) negative growth rates into the class *r* = 0 (sometimes labelled as ‘lethals’), thus artificially inflating this class [6,7,23].

Although equations (2.3) and (2.5) are classical results in population genetics [22,24], a persistent tradition in experimental evolution has been to measure relative fitness in continuously growing populations as *r*_m_/*r*_w_, that is, as a ratio of Malthusian parameters [6,7,23,25–28]. Accordingly, the selection coefficient of a mutation is often inferred from demographic parameters as *s*_r_ = *r*_m_/*r*_w_ − 1, mirroring the definition in discrete-time models from equation (2.5) [7,9,29,30]. However this measure does not directly relate to the evolutionary dynamics of mutations, even for density-independent selection with no interactions between genotypes. Notably, since *s*_r_ = *s*/*r*_w_, it would predict faster evolution per unit time in a population with the smaller growth rate *r*_w_ of the wild-type, while the actual evolutionary dynamics is the same if *s* is equal. This has important consequences for any application of these measures to questions in evolutionary biology where the magnitude of fitness effects of mutations is critical [11–13,31].

## 3. Comparing selection coefficients

The motivation for presenting results in terms of ‘relative Malthusian fitness’ in experimental evolution has been to obtain a dimensionless parameter [20], allowing for comparison between genotypes and species with different generation times. For instance, a selection coefficient measured in h^−1^ (say, for *Escherichia coli*) is not comparable to one measured in days^−1^ (say, for *Caenorhabditis elegans*). The important question for comparative purposes is how fast evolution occurs per generation. A dimensionless measurement of selection that allows comparison between studies carried out over different time scales is then3.1
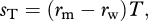
where *T* is the generation time [22, p. 178]. Electronic supplementary material, table S1, shows an overview of some of the relevant literature where fitness is measured in continuous time (that is, not from survival and fecundity per generation). In six out of nine articles, the selection coefficient per unit time *s*, or per generation *s*_T_, is not provided and cannot be estimated, because only ratios of Malthusian fitness are given, not the fitness of the wild-type *r*_w_. In only one article is *r*_w_ given, so *s*_T_ can be estimated using the generation time reported in another study for the same organism (yeast). In two cases, the selection coefficient that is provided is directly *s*_T_, since it is a difference of Malthusian fitnesses measured as growth rates per generation, not per unit time. Overall, the available data make it difficult to compare selection coefficients across species and studies.

Generation times can also vary among mutants. This may even be the main cause of genetic variation in growth rates (per unit time) for some organisms and life cycles [32]. In this case, *T* should be replaced by *T*_w_, thus measuring how fast evolution occurs relative to the generation time of the wild-type (or ‘resident’) genotype [30]. In particular, selection coefficients are often measured for unicellular organisms that reproduce by binary fission. In this case, neglecting cellular deaths, the number of generations (division events) of the wild-type per unit time is simply *r*_w_/ln2, and the generation time of the wild-type is ln2/*r*_w_. A correct measure of the dynamics of selection per generation for species reproducing by binary fission (neglecting cellular death) is then3.2
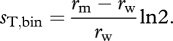


Note that if the focus of the study is fixation probabilities rather than the (deterministic) evolutionary dynamics, then *T*_m_ should be used as a scaling factor instead of *T*_w_, thus measuring the expected lifetime reproductive output of the mutant. This should affect the shape of the distribution of *s*, since a different scaling factor would be used for each mutant. None of the studies reported in electronic supplementary material, table S1, provided information about variation in generation times among mutants. Besides, comparison of expected fixation probabilities across species based solely on measures of selection coefficients from frequency changes is not to be recommended anyway, since for a given *s*, fixation probabilities can differ widely depending on how stochasticity affects offspring numbers [33].

## 4. Conclusion

Although distributions of mutation fitness effects are being measured in a growing number of organisms, lack of consistency between estimates still prevents proper comparison of selection coefficients for different species, even when selection is density and frequency independent. In particular, the use of relative Malthusian fitness results in overestimating the per-generation strength of selection (in terms of evolutionary dynamics) by a factor (ln2)^−1^ ≈ 1.44 for species that reproduce by binary fission (neglecting cellular deaths), and by an undetermined factor in other cases. It is worthwhile noting that this bias has no effect on the shape of the distribution and on the coefficient of variation of selection coefficients of mutations originating from a given genotype, an important parameter relating to the effective number of phenotypic traits under selection and to the ‘cost of complexity’ for adaptation [9,34]. Apart from this, the fitness effects of mutations across species should be re-examined in the light of the present argument, with particular attention to comparisons between species that reproduce by binary fission and others.
